# Phosphoproteomics of WHO-Priority Fungal Pathogens: Conserved Signaling Architecture, Pathogen-Specific Outputs, and Therapeutic Vulnerabilities

**DOI:** 10.3390/pathogens15070754

**Published:** 2026-07-17

**Authors:** Yuhan Ding, Chao Huang, Shuo Ning, Jingxian Liu, Yiyue Ge, Ying Chi

**Affiliations:** NHC Key Laboratory of Enteric Pathogenic Microbiology, Jiangsu Provincial Center for Disease Control and Prevention, Nanjing 210009, China; dyhroro@jscdc.cn (Y.D.); nestar1987@126.com (C.H.); ningshuo0620@163.com (S.N.); oliveppl@163.com (J.L.)

**Keywords:** phosphoproteomics, mass spectrometry, WHO-priority pathogens, antifungal resistance, signaling networks, drug target

## Abstract

Protein phosphorylation is a central post-translational modification. In pathogenic fungi, it dynamically governs morphogenesis, stress adaptation, and antifungal drug resistance. Using high-resolution mass spectrometry-based phosphoproteomics, researchers have systematically mapped phosphorylation dynamics in WHO-priority pathogens—*Candida albicans*, *Aspergillus fumigatus*, *Cryptococcus neoformans*, and the multidrug-resistant *Candidozyma auris* (formerly *Candida auris*). These studies reveal that thousands of phosphorylation events are coordinately reprogrammed in response to antifungal drug exposure, host-derived oxidative stress, and temperature shifts. Integration of available datasets suggests a “conserved-core/divergent-output” organization. Shared kinase hubs like cAMP-PKA, HOG-MAPK and calcineurin are broadly conserved across species. Downstream substrate networks, however, have diverged, producing distinct virulence outputs in each pathogen. Notably, *C. auris* remains completely uncharacterized at the phosphoproteomic level. This review provides a comprehensive synthesis of the phosphoproteomic landscape across these pathogens, and discusses how phosphoproteomic data are guiding the rational prioritization of kinases and phosphatases as next-generation antifungal drug targets—with direct implications for clinical surveillance and public health.

## 1. Introduction

Invasive fungal infections impose a disproportionate and growing burden on global health. The 2022 WHO Fungal Priority Pathogens List identifies *C. neoformans*, *C. auris*, *A. fumigatus*, and *C. albicans* as critical-priority threats [1]. Among these, *C. auris*—recently reclassified as *Candidozyma auris* based on robust phylogenetic and genomic evidence [2,3]—has been formally moved from the genus *Candida* into the newly established genus *Candidozyma*. Throughout this review, we use the abbreviated form “*C. auris*” for consistency with the majority of the primary literature cited. While these pathogens are associated with high mortality, the WHO emphasizes that a global paucity of quality surveillance data currently makes it impossible to estimate their exact attributable death toll, predominantly in immunocompromised individuals whose populations are expanding with the spread of HIV, hematological malignancy, and immunosuppressive therapy. Treatment options are restricted to three major drug classes (polyenes, azoles, and echinocandins), each with significant limitations in spectrum, toxicity, or drug–drug interaction profile. Resistance to each class is increasing: azole-resistant *A. fumigatus* is now endemic in multiple countries, echinocandin-resistant *C. albicans* is emerging in healthcare settings, and *C. auris* exhibits intrinsic or rapidly acquired resistance to all three classes simultaneously in a growing proportion of clinical isolates [4,5,6,7]. The development of next-generation antifungals with novel mechanisms of action is therefore a recognized global health priority, yet the pipeline of candidates in clinical development remains thin.

Progress toward new antifungal targets has been constrained in part by the depth of mechanistic understanding available for fungal virulence and drug resistance. While classical genetic approaches identify essential virulence factors, single-gene deletions often mask function due to network redundancy. Moreover, these methods fail to capture the dynamic, multi-pathway integration required for rapid adaptation to host stressors. Transcriptomic profiling captures changes in gene expression but, by definition, cannot detect the post-translational regulatory events that operate on timescales of seconds to minutes and are independent of transcription [8]. As a result, the signaling mechanisms by which fungi rapidly reprogram their physiology in response to immune attack, pH shifts, nutrient limitation, and antifungal drug exposure have remained substantially invisible to the tools most widely applied in fungal biology.

Protein phosphorylation is the primary mechanism by which eukaryotic cells execute rapid, reversible, and context-specific rewiring of cellular behavior. Catalyzed by protein kinases and reversed by phosphatases, the covalent addition of a phosphate group to serine, threonine, or tyrosine residues modulates protein activity, subcellular localization, stability, and interaction state, enabling a single protein to adopt functionally distinct states within seconds. In pathogenic fungi, phosphorylation cascades underpin cell cycle progression, morphogenetic switching, nutrient sensing, and stress adaptation [9]. Disruption of key phosphorylation nodes—by deletion of kinase or phosphatase genes—frequently attenuates virulence across multiple pathogens, demonstrating that phosphorylation-dependent signal transduction is not peripheral to pathogenicity but central to it. The phosphoproteome therefore represents both a mechanistic record of how fungi respond to host and drug stress, and a landscape of potential therapeutic targets.

Historically, our fundamental understanding of these phosphorylation cascades is rooted in the model yeast *Saccharomyces cerevisiae*, where the architecture of core eukaryotic signaling pathways was first delineated. These canonical networks—including the mitogen-activated protein kinase (MAPK) cascades governing osmotic and cell wall stress, the protein kinase A (PKA)/cAMP pathway directing nutrient sensing, the calcineurin pathway mediating calcium homeostasis, and the target of rapamycin (TOR) complex controlling growth and metabolism—serve as universal hubs for environmental adaptation [10]. While the core components of these signaling modules are highly conserved from non-pathogenic yeasts to human pathogens, their downstream target landscapes have undergone extensive evolutionary rewiring. Consequently, the standard pathways defined in *S. cerevisiae* are often coupled to diverse, species-specific virulence traits and drug-tolerance mechanisms in pathogens.

The development of high-sensitivity mass spectrometry combined with efficient phosphopeptide enrichment has made it possible to interrogate this landscape at a systems scale. Modern phosphoproteomic workflows identify and quantify thousands of phosphorylation sites from a single experiment [11,12,13,14], enabling a shift from hypothesis-driven, single-pathway analysis to unbiased, global profiling that captures cross-pathway signal integration, condition-specific network rewiring, and the full substrate scope of individual kinases and phosphatases simultaneously. Multi-omics integration—combining phosphoproteomic data with transcriptomics, genetics, and structural biology—further connects phosphorylation dynamics to downstream gene expression changes, phenotypic outputs, and druggable protein conformations [15]. These capabilities offer a qualitatively different kind of mechanistic insight: not simply which regulators matter, but how they are wired together, what substrates they control, and which network nodes represent the most tractable points of therapeutic intervention.

This review examines how phosphoproteomics has transformed understanding of virulence regulation, stress adaptation, and antifungal drug resistance across WHO-priority fungal pathogens. We synthesize phosphoproteomic findings from *C. albicans, A. fumigatus*, and *C. neoformans*, identify *C. auris* as the most urgent phosphoproteomic knowledge gap, and integrate cross-species data to propose a conserved-core/divergent-output framework for fungal phosphosignaling. We then consider how phosphoproteomic maps translate into drug target prioritization, network vulnerability identification, and outline priority gaps that define the field’s forward agenda.

## 2. Technical Approaches in Fungal Phosphoproteomics

### 2.1. From Fungal Cell to Global Phosphosite Map: Enrichment, Acquisition, and Quantification

The rigid fungal cell wall necessitates mechanical disruption—bead-beating with glass or zirconia beads—in the presence of phosphatase inhibitor cocktails (NaF, Na_3_VO_4_, β-glycerophosphate) to prevent post-lysis dephosphorylation [11]. After tryptic digestion, phosphopeptide enrichment by titanium dioxide (TiO_2_) chromatography, IMAC with Fe^3+^ or Ga^3+^ ions, or Fe(III)-NTA chelate resins overcomes the sub-stoichiometric abundance of phosphorylated peptides relative to the unmodified background [12,13,14]; sequential TiO_2_ and IMAC substantially increases depth and is increasingly standard [16]. Orbitrap-based LC-MS/MS with data-dependent acquisition (DDA) remains the most widely applied platform in fungal studies due to compatibility with established search algorithms (MaxQuant, Mascot, Sequest), while data-independent acquisition (DIA), which co-isolates and fragments all precursors within defined m/z windows, offers superior quantitative reproducibility for multi-condition comparisons as fungal-specific spectral libraries mature [17,18]. Phosphosite localization is assessed by probabilistic algorithms (PhosphoRS, ptmRS) with a ≥0.75 probability threshold commonly applied [19,20,21]. Quantification employs label-free quantification (LFQ), SILAC where metabolic labeling is feasible, or isobaric chemical tags (TMT/iTRAQ) for multiplexed comparisons, with phosphorylation changes normalized to total protein abundance to distinguish stoichiometric regulation from expression-level changes.

### 2.2. From Phosphosite Map to Signaling Network: Kinase–Substrate Inference and Multi-Omics Integration

**Figure 1:**
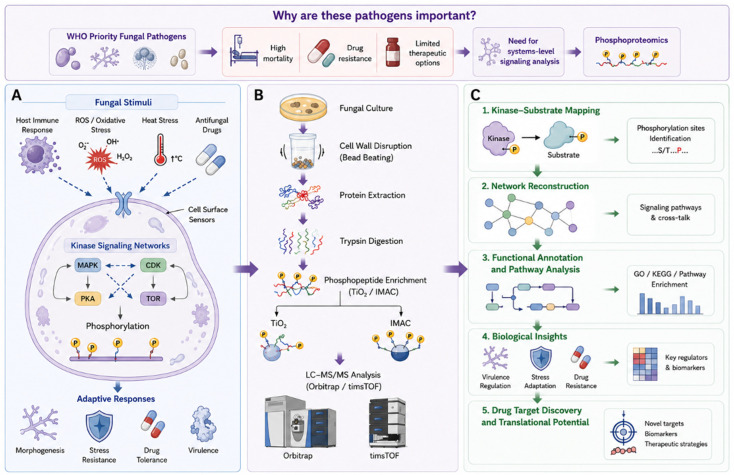
The phosphoproteomic workflow in fungal pathogens. The workflow spans from clinical priority to molecular insights and translational applications. (**A**) Fungal cells perceive diverse environmental stimuli (e.g., host immune response, heat stress, antifungal drugs) via surface sensors, triggering intracellular kinase signaling networks (MAPK, PKA, CDK, TOR) that orchestrate adaptive responses such as morphogenesis and virulence. (**B**) Technical pipeline for global phosphoproteomics, featuring mechanical cell wall disruption, phosphopeptide enrichment (TiO_2_/IMAC), and high-resolution LC-MS/MS acquisition (Orbitrap/timsTOF). (**C**) Bioinformatics and systems biology analysis for kinase–substrate mapping, signaling network reconstruction, and the prioritization of novel antifungal targets and resistance biomarkers. Figure design workflow: Initial concepts and sketches were prepared by the authors. The figure was subsequently refined through iterative prompt-based visualization and layout optimization using ChatGPT 5.6 (OpenAI). All scientific content, literature interpretation, and final verification were performed by the authors.

A phosphosite list describes what is phosphorylated; kinase–substrate inference addresses the questions of by what and why. Three complementary strategies are used to reconstruct signaling network architecture from phosphoproteomic data. First, genetic perturbation approaches compare phosphoproteomes from wild-type and kinase-deficient strains. The loss of specific phosphorylation sites following kinase inactivation enables identification of candidate kinase substrates. This strategy was instrumental in defining PKA-dependent phosphorylation networks in *C. albicans* [22] and *A. fumigatus* [23]. Second, motif-based inference uses the enriched phosphosite sequences to predict upstream kinase families (CDK, MAPK, PKA recognition motifs), providing a computational framework for assigning uncharacterized sites to known kinase families [24]; in *A. fumigatus* exposed to voriconazole stress, this approach revealed differential phosphorylation of MAPK pathway kinases including Ste20 alongside CWI and HOG pathway components [25]. Third, multi-omics integration co-analyses phosphoproteomic and transcriptomic data to discriminate phosphorylation events that regulate transcription factor activity from those with purely post-translational effects [15,26,27]. Network tools including STRING and PhosphoSitePlus support pathway enrichment and the identification of novel regulatory connections, though fungal-specific curated kinase–substrate databases remain sparse relative to mammalian resources. Together, these analytical layers convert static phosphosite inventories into dynamic signaling network reconstructions (Figure 1).

### 2.3. Current Limitations and Emerging Technologies

Several limitations constrain biological conclusions from current fungal phosphoproteomic datasets. Technically, phosphopeptides are low-stoichiometry analytes suppressed by abundant proteins during MS acquisition, requiring extensive TiO_2_ or IMAC enrichment that consumes substantial starting material—a practical barrier for slow-growing pathogens [28]. Biologically, most profiles were generated under laboratory conditions that may not recapitulate infection-relevant signaling, and single time-point snapshots cannot resolve dynamic phosphorylation trajectories. Linking phosphosites to upstream kinases remains laborious without functional validation [29].

Several emerging technologies may help overcome current limitations in fungal phosphoproteomics. TIMS-PASEF substantially improves sequencing speed and sensitivity for low-abundance phosphopeptides [30,31,32]; nanoPOTS miniaturized sample preparation reduces biomass requirements toward levels compatible with rare subpopulations or clinical specimens [33]; FAIMS-MS improves gas-phase phosphopeptide selectivity [34]; and single-cell proteomics frameworks [35,36] and spatial phosphoproteomics [37] offer, respectively, intra-population heterogeneity and subcellular localization dimensions currently inaccessible to bulk profiling. At the analytical level, AI-assisted kinase–substrate prediction [38,39] and protein energy landscape modeling [40] offer routes to connect phosphoproteomic datasets to functional and structural consequences without exhaustive experimental follow-up (Table 1).

## 3. Phosphoproteomic Landscape of *Candida albicans*

### 3.1. Baseline Phosphoproteome and Morphogenetic Signaling

Large-scale phosphoproteomic profiling of yeast-phase *C. albicans* using TiO_2_-enriched Orbitrap LC-MS/MS has identified more than 15,000 phosphorylation sites across thousands of proteins, providing the first global view of fungal phosphoregulation [24]. Motif analyses revealed enrichment of CDK-, PKA-, and MAPK-associated phosphorylation signatures. Cross-species evolutionary analysis showed that phosphosites on signaling proteins are significantly less conserved across yeast species than those on metabolic enzymes, suggesting that phosphoregulation contributes to species-specific adaptation and phenotypic divergence [41]. The morphological plasticity of *C. albicans* is central to its virulence and host adaptation. Phosphoproteomic profiling of the yeast-to-hypha transition further reveals that this process is governed by a distributed phosphorylation network, rather than a simple linear signaling cascade. Comparative studies under multiple hypha-inducing conditions, including serum, elevated temperature, and GlcNAc, identified extensive stimulus-specific phosphoregulatory programs with minimal overlap between conditions, indicating that distinct signaling architectures can converge on a common morphogenetic outcome. Network-based analysis further suggested that serine/threonine kinases dominate hyphal phosphosignaling, accounting for approximately 85% of kinase–phosphoprotein interactions, and expanded the regulatory landscape beyond the previously recognized CDK and MAPK pathways. Hypha-specific phosphorylation targets include Ssb1 (heat shock protein), Cdc19 (pyruvate kinase), TIF4631 (translation initiation factor eIF4G), and Rps4A (40S ribosomal protein) [42,43]. Subsequent multi-omics studies provided a systems-level view of the signaling circuitry underlying filamentation. Quantitative phosphoproteomic profiling of *tpk2*/*tpk2 tpk1*/*tpk1* mutants mapped hundreds of PKA-dependent phosphorylation events, and revealed that cAMP-independent signaling through the cyclin-dependent kinase Hgc1–Cdc28 and casein kinase 1 Yck2 operates in parallel with the canonical PKA–Efg1 pathway to regulate filamentous growth [22,26,27]. These findings revised earlier linear models of morphogenetic regulation and instead support a highly interconnected kinase network governing developmental transitions. Complementary phosphoproteomic analysis of cdk8Δ mutants additionally showed that the Cdk8–Mediator module gates hyphal commitment under suboptimal conditions via a Flo8-dependent pathway [44]. Phosphorylation of RNP granule components by PKA and MAPK controls post-transcriptional reprogramming during morphogenetic transitions [45], pointing to kinase-regulated RNA biology as an additional layer of therapeutic opportunity. Together, these studies established phosphorylation as a central regulatory layer controlling fungal morphogenesis and virulence-associated developmental transitions.

### 3.2. Phosphoregulatory Networks Mediating Stress Adaptation and Host Interaction

While early phosphoproteomic studies focused primarily on morphogenetic transitions, subsequent investigations revealed that phosphorylation also plays central roles in environmental adaptation and host–pathogen interactions. Integrated phospho- and proteomic analysis of H_2_O_2_-treated cells, mimicking the oxidative burst, revealed two concurrent phosphorylation events: Hog1 activates antioxidant transcription factors while ribosomal proteins and translation factors are dephosphorylated [46]. Parallel profiling of the LAMMER kinases Sky1 and Sky2 revealed distinct but complementary roles—ion homeostasis and dipeptide utilization respectively—acting through SR-like splicing factor phosphorylation [47,48]. Quantitative phosphoproteomic analysis of *C. albicans*-challenged macrophages further showed that the fungus rewires host NF-κB and MAPK substrate phosphorylation to suppress apoptosis and sustain pro-inflammatory signaling [49,50], establishing bidirectional phosphosignaling as a feature of the host–pathogen interaction.

Mechanistic connections between phosphorylation and antifungal drug stress have also emerged from these analyses. Ptc2-dependent dephosphorylation of Hsp90 within phase-separated condensates, potentiated by elevated CO_2_, destabilizes Hsp90 client kinase networks and sensitizes *C. albicans* to echinocandin-mediated cell wall stress [51], directly connecting a phosphorylation event to antifungal synergy. Proteomic characterization of the protein phosphatase Z1 (Ppz1) substrate network identified roles in cell wall stress and virulence [52], illustrating that phosphatase nodes are equally tractable targets. Collectively, the *C. albicans* phosphoproteomic record now links phosphosignaling to morphogenesis, stress adaptation, host immune evasion, and drug sensitivity—establishing a multi-condition reference framework to inform the prioritization of kinases and phosphatases for further functional investigation.

## 4. Phosphoproteomics of *Aspergillus fumigatus* and Antifungal Resistance

### 4.1. Mapping Antifungal Stress Signaling

Unlike *C. albicans,* where phosphoproteomic coverage spans multiple biological conditions, the *A. fumigatus* phosphoproteomic record is concentrated on antifungal drug stress. TiO_2_-enriched LC-MS/MS profiling of caspofungin-treated mycelia by Mattos et al. generated the first large-scale phosphosite dataset for *A. fumigatus* [53,54], which revealed coordinated activation of two MAPK pathways: the cell wall integrity (CWI) pathway centered on MpkA and the HOG pathway centered on SakA, with activation-loop phosphorylation of both kinases rising rapidly upon cell wall stress. Redundant MAPK activation may compensate for drug-induced cell wall damage, effectively limiting fungicidal efficacy and resulting in the caspofungin paradoxical effect (CPE) seen at suprathreshold echinocandin concentrations [53,54,55]. Separate phosphoproteomic characterization of the PKA catalytic subunit-dependent substrate network identified autophagy-regulatory proteins, conidial germination factors, and secondary metabolite enzymes; PKA-dependent phosphorylation of autophagy components supports carbon-source adaptation and virulence in murine aspergillosis models [23,56]. These datasets together reveal that *A. fumigatus* antifungal tolerance is not a single-pathway phenomenon but a multi-arm phosphosignaling response.

### 4.2. Phosphoregulated Drug-Tolerance Networks and Target Prioritization

Beyond pathway identification, subsequent work has focused on defining the regulatory networks that sustain adaptation and tolerance. Calcineurin phosphorylation is dynamically regulated by caspofungin treatment, and calcineurin inhibition abolishes the CPE [57], establishing calcineurin as the phosphatase hub integrating echinocandin tolerance. Mechanistically, calcineurin dephosphorylates the core septin AspB in a PP2A-dependent manner to stabilize hyphal tip integrity under cell wall stress [58] and dephosphorylates the NIMA-related kinase Kin1 at the hyphal septum [59], linking calcineurin to both structural and signaling outputs simultaneously. Comparative phosphoproteomic profiling of planktonic versus biofilm *A. fumigatus* under voriconazole revealed a biofilm-specific signature affecting cell wall composition, matrix production, efflux pump regulation, and ergosterol biosynthesis, with the phosphorylation state of transcription factors controlling cyp51A expression and efflux genes differing markedly between growth states [25]. Together, these findings organize *A. fumigatus* antifungal stress phosphosignaling around a PKA–calcineurin–MAPK network whose individual nodes—with distinct roles in echinocandin versus azole resistance—are each supported by phosphoproteomic evidence.

## 5. *Cryptococcus neoformans*: Phosphoproteomics and Pathogenicity

### 5.1. From Genetic Screens to a Reference Phosphoproteome

The *C. neoformans* phosphoproteomic record is uniquely positioned between two scales of evidence. At the genetic level, genome-wide deletion analyses of all predicted kinases (~183) and phosphatases (~114) mapped individual enzymes to virulence phenotypes—capsule production, melanin biosynthesis, thermotolerance, mating, and fluconazole susceptibility—and identified regulators without clear orthologs in model yeasts [60,61]. These screens defined the priority kinases and phosphatases most relevant to pathogenicity, but could not reveal their substrates or signaling context. Direct phosphoproteomic profiling subsequently identified more than 2400 phosphorylation sites across over 900 proteins under basal growth conditions [62], establishing the first reference phosphoproteome for *C. neoformans* and revealing a high proportion of sites on signaling proteins and transcription factors not characterized in model yeasts—providing the substrate catalog needed to interpret genetic screen hits at the phosphorylation level. Standard protein extraction protocols are severely hindered by the dense glucuronoxylomannan/galactoxylomannans (GXM/GalXM) polysaccharide capsule [63], which forms a highly viscous matrix upon cell lysis that physically traps target proteins and interferes with downstream separations. Furthermore, because mechanical disruption destroys cellular compartmentalization and releases highly active endogenous phosphatases, extraction strategies must be adapted to the encapsulation level: low-capsule strains can be directly processed with broad-spectrum inhibitors, whereas heavily encapsulated cells require ultra-low temperature methods such as liquid nitrogen grinding or strictly controlled ultrasonication or bead-beating in 8 M urea/50 mM ammonium bicarbonate. Ultimately, pairing these cold-lysis techniques with immediate TCA/acetone precipitation completely quenches enzymatic activity to maximize phosphosite recovery [62,64,65]. Systems-level phosphoproteomic analysis further revealed conserved and Cryptococcus-specific STRIPAK signaling networks, with subunit-specific phosphorylation patterns orchestrating mating responses, stress adaptation, and virulence factor regulation [66]. The combination of genetic screens and reference phosphoproteomics places *C. neoformans* in a position to accelerate condition-specific phosphoproteomic dissection of its virulence biology.

### 5.2. Phosphoproteomic Insights into Capsule and Thermotolerance

Phosphoproteomics and genetic analysis have together resolved the signaling architecture underlying three cardinal *C. neoformans* virulence traits. For capsule biosynthesis, the cAMP–PKA axis is the principal phosphorylation-dependent regulator: PKA phosphorylates downstream transcription factors including Nrg1 to govern capsule gene expression, and Nrg1 disruption attenuates virulence in murine models [67,68]. For thermotolerance at 37 °C—an absolute virulence requirement—calcineurin-dependent dephosphorylation of the transcription factor Crz1 is necessary for both thermotolerance and melanin production [69,70,71,72,73]; calcineurin substrate mapping further showed dephosphorylation of membrane trafficking regulators and nuclear export signals, providing a mechanistic basis for calcineurin inhibitor synergy with antifungals [74].

### 5.3. Network Integration and Emerging Virulence Modules

Beyond individual pathways, phosphoproteomic and genetic analysis has begun to reveal how *C. neoformans* integrates multiple stress signals. Casein kinase 2 (CK2) phosphorylates substrates across the cAMP–PKA, HOG, and calcineurin pathways simultaneously, functioning as a multi-pathway integration node for temperature, oxidative, and azole stress responses [65]. TOR complex 1 promotes thermotolerance and its inhibition attenuates virulence in rapamycin-treated models [75], while Bud32 kinase regulation of iron homeostasis connects phosphosignaling to nutrient acquisition under host conditions [76]. Phosphoproteomic profiling of *C. neoformans*-infected host cells revealed global reprogramming of host MAPK, PI3K, and Src signaling [77], and the adenylyl cyclase Cac1 was found to harbor a PP2C phosphatase domain functionally separable from its cyclase activity [78], adding an unexpected layer of self-regulatory signal integration within the cAMP pathway. A unique glucosamine-triggered cell wall response controls chitin-to-chitosan conversion through phosphorylation-dependent signaling, a virulence-associated modification absent from non-pathogenic yeasts [79]. Comprehensive phosphoproteomic datasets under host-relevant stress conditions nonetheless remain sparse compared with *C. albicans* and *A. fumigatus*; the reference phosphoproteome [62] and STRIPAK analysis [66] have only begun to close this gap.

## 6. *Candidozyma auris* (Formerly *Candida auris*): Phosphorylation Signaling and Multidrug Resistance

### 6.1. Inferences and Limitations of Non-Proteomic Data

*C. auris* presents the starkest contrast in the WHO-priority pathogen phosphoproteomic landscape: despite its intrinsic or rapidly acquired resistance to all three major antifungal classes and its clonal spread through healthcare networks globally [4,80,81], no large-scale phosphorylation profiling study exists for this organism. Traditional genetic experiments have established the functional roles of core stress hubs—Hog1, calcineurin, and PKA—in *C. auris* [82,83,84,85,86,87], but whether these pathways are functionally reprogrammed at the phosphorylation level in response to drug stress or host conditions is entirely untested. Reviews of *C. auris* resistance mechanisms have implicated ERG11 mutations, FKS hot-spot alterations, and efflux pump overexpression [5]—each likely under phosphorylation-dependent transcriptional control, but without direct evidence. Carbonic anhydrase Nce103 mediates skin tropism and antifungal resistance [88], and PDR16 gene dosage modulates azole susceptibility through phosphatidylinositol transfer [89]; the phosphorylation basis of both mechanisms remains unexplored. The absence of phosphoproteomic data means that the signaling logic underlying *C. auris* multidrug resistance—the defining clinical feature of this pathogen—is currently inaccessible.

### 6.2. Priorities and Translational Potential

**Figure 2:**
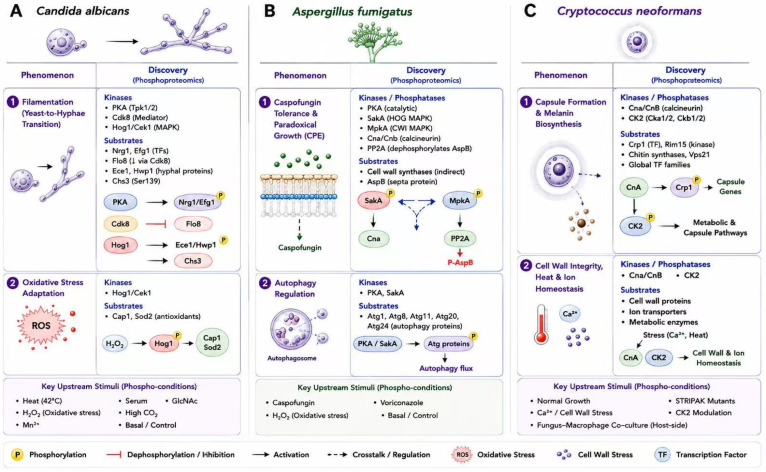
Biological phenomena decoded by phosphoproteomics. Phosphoproteomic studies have revealed how environmental and host-associated cues are translated into signaling networks that govern fungal pathogenicity. In *Candida albicans* (**A**), phosphorylation regulates morphogenesis and oxidative stress adaptation; in *Aspergillus fumigatus* (**B**), it underpins caspofungin tolerance and autophagy-associated responses; and in *Cryptococcus neoformans* (**C**), it coordinates capsule-associated functions, stress adaptation, and virulence. Together, these findings illustrate how phosphoproteomics links upstream stimuli to signaling hubs and biologically relevant phenotypes. Figure design workflow: Initial concepts and sketches were prepared by the authors. The figure was subsequently refined through iterative prompt-based visualization and layout optimization using ChatGPT 5.6 (OpenAI). All scientific content, literature interpretation, and final verification were performed by the authors.

The immediate priority is a reference phosphoproteomic atlas spanning multiple *C. auris* clinical clades under antifungal stress, biofilm, and host-mimicking conditions. Such a dataset would identify the phosphorylation networks underlying multidrug resistance, enable clade-specific target prioritization, and—by direct comparison with *C. albicans* phosphoproteomic data—distinguish resistance mechanisms that are conserved across *Candida* species from those specific to *C. auris*. Phosphoproteomic signatures could ultimately stratify clinical isolates by mechanistic resistance profile, supporting targeted therapeutic decisions and longitudinal surveillance of resistance evolution across healthcare networks [80,81]. Generating this foundational dataset is the single most urgent phosphoproteomic priority in clinical mycology (Figure 2, Table 2).

## 7. A Conserved-Core, Divergent-Output Architecture of Fungal Phosphosignaling

Integrated datasets suggest a “conserved-core/divergent-output” model for fungal signaling. In this framework, a few kinase and phosphatase hubs remain sequence-conserved, while their downstream substrate networks have rewired to produce pathogen-specific virulence outputs. This conserved-core/divergent-output architecture has not been formally demonstrated by a unified comparative experiment—existing datasets were generated by different laboratories under different conditions.

### 7.1. Conserved Kinase and Phosphatase Hubs

**Figure 3:**
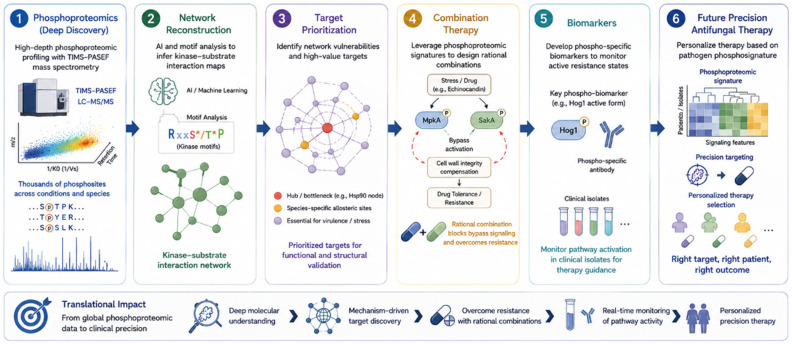
From phosphoproteomic maps to therapeutic vulnerabilities. The six-step pipeline illustrates the integration of molecular discovery and clinical application. (**1**,**2**) High-depth phosphoproteomic profiling and AI-driven network reconstruction define the landscape of kinase–substrate interactions. (**3**,**4**) Target prioritization identifies signaling hubs and bypass activation pathways, enabling the design of rational combination therapies to overcome drug resistance. (**5**,**6**) The development of phospho-specific biomarkers and pathogen signatures facilitates real-time monitoring and personalized therapy selection. The overall workflow (bottom panel) transitions from deep mechanistic understanding to the “right target, right patient, right outcome” paradigm in clinical mycology. Figure design workflow: Initial concepts and sketches were prepared by the authors. The figure was subsequently refined through iterative prompt-based visualization and layout optimization using ChatGPT 5.6 (OpenAI). All scientific content, literature interpretation, and final verification were performed by the authors.

The fundamental backbone of fungal phosphosignaling is ancient and highly constrained. The cAMP–PKA axis, the HOG–MAPK pathway, and calcineurin are each represented across all three pathogens with conserved kinase recognition motifs and conserved roles in core stress response functions (Figure 3). Crucially, these core regulatory cascades share near-identical catalytic topologies with the non-pathogenic model yeast *Saccharomyces cerevisiae*, reflecting a universal baseline for fungal cell survival.

Calcineurin, for example, is required for various functions in *C. albicans* [73,74], *A. fumigatus* [57], and *C. neoformans* [70,71], and its inhibition attenuates virulence in all three organisms—the clearest current example of a phosphoregulatory node that is simultaneously pan-fungal in conservation and pan-fungal in therapeutic relevance. Hsp90 represents a second conserved hub: its phosphoregulatory interactions with client kinases govern morphogenesis, biofilm formation, and echinocandin resistance across *Candida* species [91,92,93,94,95], and Ptc2-dependent dephosphorylation of Hsp90 within phase-separated condensates directly sensitizes *C. albicans* to echinocandin treatment [51]. The structural alignment of these hubs between *S. cerevisiae* and critical pathogens suggests that the evolutionary constraint on kinase–substrate recognition chemistry is immense; the underlying catalytic machinery remains locked across hundreds of millions of years of divergence.

### 7.2. Divergent Phosphoproteomic Outputs

Against this conserved backbone, the downstream phosphoproteomic outputs are clearly divergent, showcasing a profound evolutionary rewiring where standard environmental responses are repurposed into lethal pathogenicity traits (Table 3).

The cAMP–PKA axis exemplifies this shift from non-pathogenic homeostasis to virulence. In *S. cerevisiae*, PKA signaling predominantly monitors nutrient availability to control gene expression and metabolic adaptation [10,96]. In stark contrast, human pathogens have decoupled or expanded this network to govern host invasion: it controls hyphal morphogenesis in *C. albicans* via Efg1, Flo8, and Ume6 [22], yet regulates capsule biosynthesis in *C. neoformans* by targeting distinct transcription factors [60,67,68]; and in *A. fumigatus*, the PKA substrate network is enriched for autophagy regulators and secondary metabolite biosynthetic enzymes linked to metabolic flexibility and virulence [23,56].

A similar logic applies to HOG–MAPK signaling. Originally characterized in *S. cerevisiae* as a dedicated mechanism to survive high osmolarity (such as salt stress) [10,97], the pathway has been rewired in pathogens to combat host-imposed defenses and clinical interventions: in *C. albicans,* Hog1 orchestrates the antioxidant transcriptional response to macrophage-derived oxidative stress [46]; in *A. fumigatus*, SakA is co-activated with MpkA during echinocandin treatment, contributing to the caspofungin paradoxical effect [53]; and in *C. neoformans*, the HOG pathway contributes to virulence and differentiation [69]. The same conserved kinase hub thus controls morphogenetic switching, polysaccharide capsule production, and metabolic adaptation in three different pathogens—each a cardinal virulence strategy adapted to a distinct host niche.

### 7.3. Pathogen-Specific Structural and Network Innovations

Species-specific phosphoregulatory innovations are superimposed on this divergent-output layer: LAMMER kinases Sky1/Sky2 and the Cdk8–Mediator axis are *C. albicans*-specific regulatory modules [44,47,48], STRIPAK signaling is a *C. neoformans*-specific network architecture [66], and the calcineurin–septin–Kin1 axis is characterized primarily in *A. fumigatus* [57,58,59]. These species-specific nodes, lacking clear functional orthologs in other pathogens or benign yeasts, represent drug targeting opportunities with inherently narrow-spectrum profiles but exceptionally high potential for pathogen selectivity.

## 8. From Phosphoproteomic Maps to Therapeutic Vulnerabilities

### 8.1. Mechanistic Target Identification and Selectivity Assessment

Phosphoproteomics elevates drug discovery from simple genetic essentiality to high-resolution mechanistic insights by identifying which hubs are actively signaling under precise drug or host stresses. Genetic screens previously established hubs like calcineurin as virulence-essential [60,61,70]; phosphoproteomic substrate mapping in *C. neoformans* [74] and *A. fumigatus* [57,58,59] subsequently clarified the mechanistic basis for this essentiality, defining how to achieve therapeutic phenotypes with reduced toxicity. Multi-kinase inhibitor profiling combined with phosphoproteomic selectivity assessment has identified anti-*C. albicans* compounds with preferential activity over human kinases [98,99], providing proof-of-concept that phosphoproteomic substrate signatures can replace or supplement biochemical panel screening for selectivity determination. Together, comprehensive substrate mapping, biophysical characterization of allosteric sites, and in-depth selectivity profiling offer new avenues to overcome a long-standing bottleneck in fungal kinase drug discovery—the high structural conservation of ATP-binding pockets between fungal and human kinases, which severely constrains the selectivity of active-site-directed inhibitors.

A separate but equally pressing challenge is functional interpretation: even in well-studied systems such as the human phosphoproteome, over 90% of identified phosphosites lack functional annotation [38]; in fungi, where the dataset is considerably smaller, the annotation gap is even more pronounced. Bridging this gap requires transitioning from data generation to prioritized functional validation. Integrating mass spectrometry datasets with AI-assisted kinase–substrate inference tools [38,39] and protein energy landscape modeling [40] offers a scalable pipeline to fast-track candidates into structure-based inhibitor design.

### 8.2. Network Vulnerability, Combinatorial Design, and Resistance Biomarkers

A recurring finding across all three pathogens is that antifungal drug tolerance is not mediated by a single pathway but by converging, redundant phosphosignaling networks. In *A. fumigatus*, the echinocandin paradoxical growth effect requires co-activation of both the MpkA (CWI) and SakA (HOG) pathways [53], and calcineurin inhibition alone abolishes it [57]; no single-target intervention replicates calcineurin inhibition’s effect on the CPE. In *C. albicans*, Hsp90–calcineurin interactions sustain echinocandin tolerance through a client kinase network that is disrupted by Ptc2-mediated dephosphorylation [51,95]. Phosphoproteomic network topology maps these exact intersection points. Rather than relying on empirical drug screening, this approach identifies which nodes, when co-targeted, are topologically guaranteed to collapse compensatory responses.

Beyond combination design, these activation topologies offer potential as functional biomarkers of clinical resistance. While genotype-based profiling flags resistance mutations, it cannot report if those mechanisms are actively engaged. Monitoring activation-state markers of key resistance pathways (calcineurin, HOG, CWI, PKA) could provide direct phenotypic readouts. The immediate prerequisite is establishing which specific phosphorylation events most reliably distinguish drug-tolerant from sensitive states in well-characterized collections.

### 8.3. Coverage Gaps and the Candida auris Priority

The most immediately visible gap is coverage: *C. albicans* has the deepest phosphoproteomic record (~15,000 sites) [24,43], *C. neoformans* is mapped at lower depth (~2400 sites, predominantly under basal conditions) [62], *A. fumigatus* coverage is concentrated on antifungal drug stress [23,53,54], and *C. auris* has no published phosphoproteomic dataset at all. Even for the best-characterized species, profiling under host-relevant conditions (body temperature, nutrient limitation, immune challenge, in vivo infection) remains sparse. The majority of existing datasets were generated under standard laboratory growth conditions. Expanding coverage to include infection-mimicking conditions, multiple clinical isolates and clades, and time-resolved sampling following drug or host-stress exposure is a prerequisite for connecting phosphoproteomic discoveries to clinical biology. For *C. auris* specifically, a reference phosphoproteomic atlas spanning its five major clinical clades [100] under antifungal stress and biofilm conditions would simultaneously close the most urgent clinical gap and enable direct comparison with *C. albicans* data to distinguish conserved from *C. auris*-specific resistance mechanisms.

## Figures and Tables

**Table 1:** Emerging methodological directions.

Technology	Main Application	Potential Value in Fungal Phosphoproteomics	Current Readiness	Reference
TIMS-PASEF	Deep phosphosite identification	Improved detection of low-abundance signaling proteins	Mature	[30]
DIA-MS/ DIA-NN	Quantitative phosphoproteomics	High reproducibility across multiple conditions	Mature	[17,18]
nanoPOTS	Low-input phosphoproteomics	Analysis of rare fungal populations and clinical samples	Emerging	[33]
FAIMS-MS	Gas-phase ion filtering	Increased phosphopeptide selectivity	Emerging	[34]
Single-cell Proteomics	Cellular heterogeneity analysis	Investigation of fungal phenotypic heterogeneity	Experimental	[35,36]
Spatial Phosphoproteomics	Host–pathogen interface mapping	Analysis of signaling during infection	Early-stage	[37]
Protein Energy Landscape Modeling	Large-scale analysis of conformational fluctuations and stability	Understanding how phosphorylation alters local stability and conformational dynamics	Early-stage	[40]
AI-assisted Kinase Prediction	Kinase–substrate assignment	Reconstruction of fungal signaling networks	Emerging	[38,39]

**Table 2:** Representative omics and phosphoproteomic studies in WHO-priority fungal pathogens.

Pathogen	Condition	MS Platform	Key Findings	Reference
*C. albicans*	Baseline Phosphoproteome (yeast phase)	Orbitrap, TiO_2_ + IMAC	>15,000 phosphosites (80% Ser, 18% Thr, 2% Tyr); CDK, PKA, MAPK motifs enriched; Mediator complex phosphorylation by Cdk8.	[24]
	Hyphal morphogenesis	Orbitrap, TiO_2_, LC-MS/MS	60, 20, 53 unique phosphoproteins per condition; Ser/Thr/Tyr distribution; interaction network with 19 kinases.	[43]
	PKA null mutant (*tpk2*/*tpk2 tpk1*/*tpk1*)	LC-MS/MS, TiO_2_	Global PKA substrate network; regulation of hyphal growth, stress response, and virulence.	[22]
	Multi-omics (cAMP-independent mechanisms)	Orbitrap, TiO_2_	Identified Hgc1-Cdc28 and Yck2 pathways parallel to PKA; revised model of morphogenesis.	[26]
	cdk8Δ mutant (hyphal repression)	Orbitrap, TiO_2_	Cdk8 represses hyphal growth via Flo8-dependent pathway; dozens of Mediator substrates.	[44]
	Oxidative stress (H_2_O_2_, mimicking macrophage burst)	Orbitrap, IMAC	Hog1 activates antioxidant TFs; ribosomal proteins dephosphorylated to reduce misfolded proteins.	[46]
	CO_2_-dependent Echinocandin Sensitization	Orbitrap, Co-IP	Elevated CO_2_ promotes Ptc2-dependent dephosphorylation of Hsp90 within condensates, destabilizing client kinase networks and enhancing echinocandin efficacy.	[51]
	Manganese Stress	LC-MS/MS	Mn^2+^-modulated phosphoproteome identifies novel ion homeostasis and cell wall kinase substrates.	[90]
	Phosphatase Z1 (Ppz1) Substrate Network	LC-MS/MS	Broad Ppz1 substrate network implicated in cell wall stress responses, ion homeostasis, and virulence.	[52]
*A. fumigatus*	Caspofungin Exposure (CPE)	Orbitrap, TiO_2_	Co-activation of MpkA (CWI) and SakA (HOG) MAPKs; explains paradoxical growth effect.	[53,54]
	Voriconazole-treated Biofilms	LC-MS/MS, TiO_2_	Biofilm-specific phosphosignature; predicted upstream kinases (CkA2, Ste20, NimA) for azole resistance.	[25]
	PKA catalytic subunit-dependent	LC-MS/MS, TiO_2_	PKA substrates span autophagy regulators, conidial germination factors, and secondary metabolite enzymes.	[23,56]
*C. neoformans*	Global Phosphoproteome (basal)	Orbitrap, TiO_2_	>2400 phosphosites on >900 proteins; high proportion of signaling proteins and TFs.	[62]
	STRIPAK Complex (systems-level)	Orbitrap, Co-IP MS	Conserved and subunit-specific phosphorylation patterns; mating, stress, virulence regulation.	[66]
	Casein kinase 2 complex	LC-MS/MS	CK2 integrates cAMP-PKA, HOG, and CWI pathways; regulates temperature, oxidative, azole stress.	[65]
	Calcineurin Substrate Mapping	LC-MS/MS, TiO_2_	Identified dephosphorylation of membrane trafficking regulators, nuclear export signals, and Crz1.	[74]
*C. auris*	--	--	No large-scale phosphoproteomic study available.	

“--” means no research data available yet.

**Table 3:** Conserved kinase hubs and species-specific phosphoregulatory modules identified in fungal phosphoproteomic studies.

Target Name	Class	Species	Virulence Function	Reference
PKA	cAMP-dep. Kinase	**Conserved** *Pan-Fungal*	Morphogenesis; virulence gene expression; capsule (*C. neoformans*); Nrg1 regulation	[22]
Hog1/SakA	MAPK (p38-like)	**Conserved** *C. albicans*, *A. fumigatus*	Oxidative stress; HOG pathway; CPE in *A. fumigatus*; thermotolerance in *C. neoformans*	[46,53]
Calcineurin (CnA/CnB)	Ser/Thr Phosphatase	**Conserved** *Pan-Fungal*	Thermotolerance 37 °C; drug tolerance (azoles, echinocandins); Crz1 dephosphorylation	[69,73]
MpkA/Mkc1	Cell Wall Integrity MAPK (Slt2-like)	**C** **onserved** *C. albicans,* *A. fumigatus*	Echinocandin tolerance; cell wall remodeling; paradoxical effect via Hog1 crosstalk	[53,54]
CDK1/2	Cyclin-dep. Kinase	**Conserved** *Pan-fungal*	Cell cycle progression; G1/S and G2/M checkpoint control; morphogenesis in *C. albicans*	[24]
Casein Kinase 2	Ser/Thr Kinase	**Conserved** *Pan-fungal*	Ribosome biogenesis; stress adaptation; biofilm; TF phosphorylation in *A. fumigatus*	[54]
Sky1/Sky2	LAMMER Kinase	**Specific** *C. albicans*	SR splicing factor phosphorylation; alternative splicing under host stress; systemic virulence	[48]
Gin4/Hsl1	Septin-assoc. Kinase	**Specific** *C. albicans*	Septin ring assembly; yeast-to-hypha transition; polarized tip growth	[43]
Cdk8 (Ssn3)	Cyclin-dep. Kinase	**Specific** *C. albicans*	Represses hyphal growth via Flo8; phosphorylates Mediator subunits (Med3, Med4, Med8, Med9, Med15, Med13)	[44]
Ptc2	PP2C Phosphatase	**Specific** *C. albicans*	Dephosphorylates Hsp90 within phase-separated condensates; CO_2_-dependent	[51]
Bud32	Kinase (EKC/KEOPS complex)	**Specific** *C. neoformans*	Regulates iron homeostasis; links phosphorylation to nutrient acquisition under host conditions	[76]
STRIPAK (Far3/Far7/Pph21)	Signaling scaffold	**Specific** *C. neoformans*	Orchestrates mating, stress adaptation, and virulence factor regulation via subunit-specific phosphorylation	[66]

## Data Availability

No new data were created or analyzed in this study. This manuscript is a review article; all data discussed are available in the original studies cited herein. Data sharing is not applicable to this article.
